# Basketball specific agility: A narrative review of execution plans and implementation effects

**DOI:** 10.1097/MD.0000000000037124

**Published:** 2024-02-09

**Authors:** Weicheng Li, Yongfeng Liu, Jiaxin Deng, Tong Wang

**Affiliations:** a School of Sports Training, Chengdu Sport University, Chengdu, Sichuan, China.

**Keywords:** agility, athletes, basketball, training methods

## Abstract

This systematic review and evaluation aim to comprehensively overview current international advanced basketball specialized agility training methods. The primary objective is to analyze and synthesize existing literature, offering insights and guidance to enhance agility training levels specifically tailored for basketball players. Methods involved a systematic literature search using keywords like “Basketball,” “Agility,” and “Training” in major databases (PubMed, Web of Science, and EBSCO), covering studies from 2010 to 2022. Inclusion criteria focused on studies addressing advanced agility training methods for basketball players. Data extraction and analysis were conducted to identify key trends and outcomes. A total of 563 articles were initially identified, and after reviewing titles, abstracts, and full texts, 20 articles were ultimately selected, excluding those with inconsistent outcome measures or unavailable full texts. The findings suggest that plyometric training, comprehensive speed training, and equipment-assisted training methods (SSG, TRX, Bulgarian ball, etc) have demonstrated effectiveness in improving agility indicators in basketball players.

## 1. Introduction

Basketball, as a global sport, not only showcases its appeal at the professional level but also deeply influences the daily lives of schools and communities. As a high-intensity sport, basketball places exceptionally high demands on the overall qualities of athletes. Outstanding basketball players not only require excellent basketball-specific skills but also need to achieve comprehensive development in physical fitness. Robust physical fitness includes outstanding speed, strength, agility, flexibility, and balance, among other qualities. In the context of basketball, agility involves various aspects, including reaction speed, precision of movement, quick changes of direction, and rapid decision-making abilities. Therefore, agility is a highly complex and defining athletic quality in basketball. Assessment and training typically involve comprehensive discussions on speed, strength, reaction time, and other related qualities.^[1–3]^ However, there is currently no consensus in the field of sports science regarding the definition of agility. According to scholarly discussions, agility is generally defined as the speed of response to stimuli or rapid changes of direction ability. Many fitness coaches believe that improving linear acceleration can enhance the ability to change direction quickly, but empirical evidence has shown no support for this viewpoint. BAKER^[4]^ used the Illinois Agility Test and a 20-meter sprint to compare and assess the athletic performance of elite and developmental league-level rugby players. The results indicated equal linear sprinting speeds between the 2 groups, but elite athletes performed better in change of direction tests. This demonstrates that improving linear acceleration does not enhance change of direction ability and, consequently, does not improve agility in basketball players. Similarly, many fitness coaches believe that resistance strength training is closely related to linear sprinting performance and can improve change of direction ability, as mentioned earlier. However, due to their distinct physical qualities, linear sprinting and changing direction running are not correlated.^[5]^ Hence, it is inappropriate to extrapolate that strength training methodologies tailored for sprinting are equally applicable to sports such as basketball, characterized by frequent and dynamic changes in direction. Consequently, there is a pressing need for comprehensive research on agility training methodologies specifically designed for basketball. However, the correlation between agility training techniques in basketball and the enhancement of agility metrics in basketball players remains inadequately explored. Consequently, this study endeavors to investigate the impact of agility training methods on the agility of basketball players, discern the effectiveness of diverse training approaches on the agility metrics of basketball players, and thereby contribute theoretical underpinnings for the formulation of personalized training regimens for basketball players.

## 2. Methods

### 2.1. Search strategy

The search queries were constructed using Boolean logic to precisely retrieve relevant literature from PubMed, Web of Science, and the EBSCO database. The literature considered spans from 2010 to 2022. The search queries are outlined as follows: for Web of Science, TS=(“agility” or “flexible” or “nimble” or “agile”) and (“basketball”) and (“training”); for PubMed and EBSCO databases, searching for keywords “agility” or “flexible” or “nimble” or “agile” and “basketball” and “training.” Following the literature search, a double-blind approach involving 2 independent researchers will be employed for the inclusion and exclusion process. Each library of documents will be imported into EndnoteX9 literature management software, where the initial deduplication will be performed using the software integrated system. Further scrutiny will involve reading titles and author years, followed by a preliminary screening of papers based on titles and abstracts. Subsequently, the selected articles from this screening will be downloaded, and in cases of discordant extractions by the 2 researchers, a third researcher will be consulted to deliberate on the inclusion of differing articles.

### 2.2. Inclusion and exclusion criteria for literature

#### 2.2.1. Inclusion criteria.

1. Experimental Studies.
2. Basketball Agility Metrics
3. The subjects were basketball players

#### 2.2.2. Exclusion criteria.

1. Non-English Paper.
2. Incompatible Intervention Methods: Studies with intervention methods that do not align with the research focus will be excluded.
3. Sports Other Than Basketball: Literature focusing on sports other than basketball will be excluded.
4. Unavailability of Full Text: Studies for which the full text cannot be obtained will be excluded.

### 2.3. Data extraction and analysis

Data extraction for the research was conducted independently by 2 authors(Li Weicheng and Deng Jiaxin).The gathered data was imported into EndnoteX9 software, encompassing the extraction of essential literature details such as authorship and publication year. Additionally, participant information, including age, gender, and sample size, was collected. Details on intervention measures, such as intervention type, exercise frequency, and duration, were also extracted. Lastly, outcome measures information, covering agility-related assessment scales and tests, was included. This meticulous process ensures a comprehensive and accurate compilation of data for further analysis.

## 3. Results

### 3.1. Literature search results

**Figure 1.**
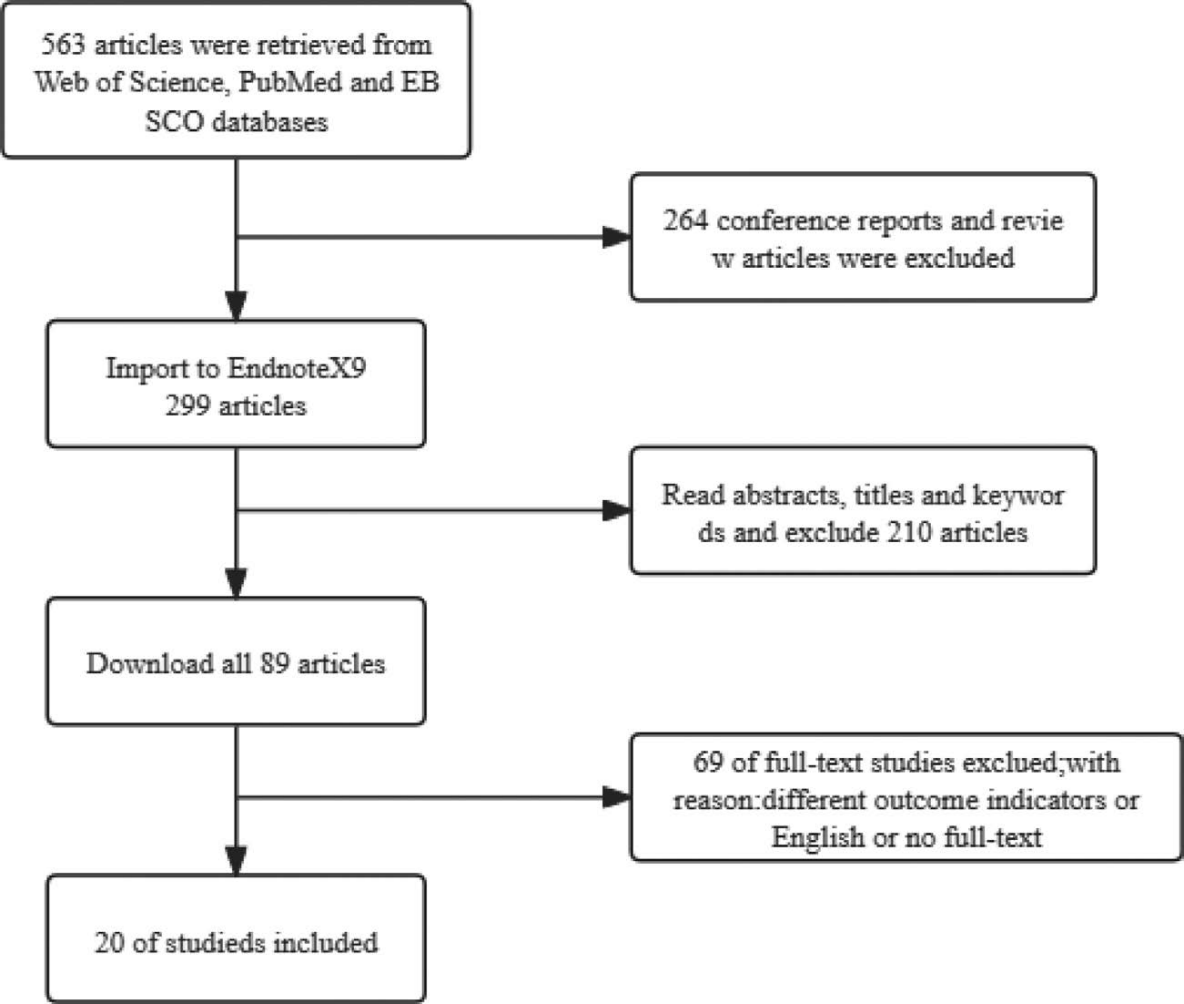
Flowchart of literature search and selection.

The results of the search in PubMed, Web of Science and EBSCO databases were 563. Finally, 20 articles on basketball agility were included in the systematic review. The selection process is shown in Figure 1.

### 3.2. Inclusion of basic literature information

A total of 20 articles were included in this study, including 22 studies, which were designed as RCT studies, cohort studies, etc. The sample size of each study was between 10 and 58. The article reports the sports style, frequency, cycle and agility quality evaluation scale of basketball players. The exercise methods are mainly plyometric training and comprehensive training. The exercise cycle ranges from 2 weeks to 10 weeks, and the exercise frequency is basically 1 to 6 times per week. The basic characteristics of the included literature are shown in Table 1 below.

**Table 1:** Summary of the effect of different exercise types on agility in basketball player.

First author, yr	Sample size	Measure	Period/wk	Frequency	Outcome indicator	Result
Delextrat, 2014	24	SSG	6	2 times/wk	Defensive agility	+4.5, *P* < .05*
Abbas, 2017	23	PCT		2 times/wk	IAT	*R* = 0.435,*P* < .05*
Artan, 2019	20	PT	4	1 times/wk	IAT	*P* = .004*
Asadi, 2012	16	PT	6	2 times/wk	IAT	+7%
Christopher, 2013	10	HRW		1 time	IAT	*P* = .074
ZEMKOVA, 2010	34	ABT	6	5 times/wk	Agility test	1770.4 ± 90.2–1667.5 ± 91.4 ms
Ersan, 2022	32	SSG	6	3 times/wk	T-Drill test	12.56 ± 0.85–11.84 ± 0.73
Gokmen, 2020 (1)	12	PTS	6	3 times/wk	Agility test	15.61 ± 1.42–14.12 ± 1.36
Gokmen, 2020 (2)	12	PTW	6	3 times/wk	Agility test	15.92 ± 1.87–14.82 ± 1.56
Hamid, 2012 (1)	18	PTA	8	3 times/wk	Agility T-Test	−5.90%*
Hamid, 2012 (2)	18	PTL	8	3 times/wk	Agility T-Test	−6.08%
HANY, 2017	10	Bulgarian-Bag training	8	4 times/wk	Agility T-Test	4.53 ± 0.14–4.31 ± 0.15
Zeng, 2021	19	SSG	4	2 times/wk	MAT	+7.2%, d = 1.7
Larissa, 2017	25	Circle training	8	3 times/wk	Endurance Shuttle	−3.80 s, +6.66%
Klusemann, 2012	13	Online video–based training	6	2 times/wk	Agility Test	−1.7 ± 2.4%
Mehmet, 2020	26	Speed training	8	3 times/wk	Agility Test	9.57 ± 0.35– 9.45 ± 0.10
Michal, 2013	12	PT	6	5 times/wk	T-Drill test	9.35 ± 0.49– 9.42 ± 0.40
Özgür, 2018	20	Suspension training(TRX)	6	2 times/wk	T-Drill test	*P* < .05*
Özhan, 2016	20	Step-aerobic exercises	8	3 times/wk	Flexibility	*P* < .05*
Pedro, 2017	58	Contrast training	10	3 times/wk	T-test	14.57 ± 1.29*
Selvam, 2014	30	PT	2	6 times/wk	Agility T-Test	16.75 ± 2.49–16.75 ± 2.49*
Chen, 2018	30	Shuttle-run training	8	3 times/wk	Agility T-Test	9.7 ± 0.7– 9.9 ± 0.5

ABT = agility-balance training, COD test = change of direction ability test, HRW = heavy resistance warm-up treatment, IAT = Illinois Agility Test, MAT = modified agility test, PCT = Postural control training, PT = plyometric training, PTA = plyometric training aquatic, PTL = plyometric training land, PTS = plyometric training sand, PTW = plyometric training wood, SSG = small-side game.

* Values are significantly different (*P* < .05).

## 4. Discussion

### 4.1. The impact of different training modalities on agility in basketball

#### 4.1.1. The impact of plyometric training on agility in basketball.

Plyometric training is widely recognized as a beneficial approach to improving agility in basketball. Key methods include jump training, resistance training, and speed training. Plyometric training is also referred to as the stretch-shortening cycle (SSC). It involves stretching and shortening of elastic tissues, transferring elastic potential energy to elastic structures for storage. During the eccentric contraction phase, elastic potential energy is released, reinforcing concentric contractions, thereby enhancing overall physical performance in the body.^[6,7]^ Additionally, during the process of muscle stretching, proprioceptors perceive the tension in the muscles. Subsequently, these proprioceptive signals are transmitted to the spinal cord, recruiting motor units and motor neurons. This process contributes to the enhancement of overall physical performance in the body.^[8]^ Therefore, to achieve maximum effectiveness in enhanced training, it is essential to ensure that the exercise load corresponds appropriately to the intensity.^[9]^

Artan^[10]^ suggest that comprehensive jumping exercises such as Front Cone Hops and Lateral Cone Hops over a 4-week period can enhance the agility of basketball players. Through various rapid jumping exercises, the nervous system rapidly transmits signals to muscle groups, prompting rapid muscle fiber contraction. This, in turn, enhances muscle explosive power, strengthening the agility of basketball players. Özen,Gökmen^[11]^ suggest that modifying the training conditions on the basis of Plyometric training can more effectively improve agility in male basketball players. Conducting Plyometric training in a sandy environment for 6 weeks is shown to alter the agility of athletes more effectively. This is attributed to the instability factors present in the sandy environment, leading to a broader range of motion and increased involvement of various muscle groups, thereby enhancing core stability and explosive power.^[12]^ Similarly, Hamid^[13]^ believes that underwater Plyometric training can enhance the agility of basketball players. The buoyancy in the aquatic environment can reduce landing forces, contributing to improved agility in athletes.^[14]^ Additionally, the buoyancy effect in the aquatic environment provides for faster concentric movements, resulting in greater power output and, consequently, enhanced agility in basketball players. Therefore, the combination of underwater environments and Plyometric training appears to be a complementary and effective training approach.^[15,16]^ In summary, Plyometric training has the potential to improve agility in basketball players, and its positive impact is further augmented by diverse adjustments to training conditions.

#### 4.1.2. The influence of speed training on agility in basketball.

Speed is one of the key factors in basketball, as athletes aim to move rapidly from 1 position on the basketball court to another and execute actions with maximum intensity within a short time frame,^[17]^ It encompasses quick reaction capability, explosive power, and the rapid mobilization of the nervous system.^[18]^ Speed training is focused on enhancing an athlete ability to move quickly, aiming to improve their starting speed, acceleration, change of direction skills, reaction time, and overall agility. The training encompasses linear acceleration, multidirectional movements, and rapid transitions between actions.^[1]^ Mehmet^[19]^ suggests that an 8-week speed training program, incorporating multiple sets of 10 to 60 m sprints at intensities ranging from 80% to 100%, can significantly enhance the agility of basketball players. This high-intensity sprinting is expected to increase the quantity and efficiency of fast-twitch muscle fibers, thereby improving agility, explosive power output, and rapid reaction capabilities. Concurrently, it is anticipated to elevate cardiovascular health, enhance the pumping capacity of the heart, and improve the elasticity of the vascular system, consequently improving the endurance and recovery capacity of athletes.

Larissa^[20]^suggests that significant improvement in agility (6.66% reduction in time, from 3.80 seconds) can be achieved through a 4-week, 4-exercise circuit training program, which includes jump, long-horse, running and dribbling, and reversing exercises. Compound exercises contribute to the overall development of athletes, with jumping and running exercises enhancing rapid muscle contraction and lower limb strength. Dribbling and reversing involve quick changes in movement, contributing to adaptive improvements in the nervous system. Running and reversing activities enhance cardiovascular function, strengthen athlete endurance, and contribute to improved posture control during dribbling direction changes and jumping movements. This helps enhance the athletes’ overall coordination and balance, enabling them to maintain a high level of agility during competitions.

Moreover, Christopher^[21]^ suggests that pre-training heavy resistance warm-up can improve the speed performance of basketball players. Joint flexibility is another crucial factor influencing speed training; if muscle groups are too tense, maximum energy cannot be exerted throughout the entire movement, thereby inhibiting agility performance. Therefore, heavy resistance warm-up training is essential for agility in basketball. Heavy resistance warm-up exercises, through different intensity squat movements, result in an increase in muscle temperature and alterations in the viscoelastic behavior of the muscle-tendon unit,^[22]^ enhancing athletes’ speed performance is a key factor in improving agility.

#### 4.1.3. The impact of diversified training methods on agility in basketball.

In today intense basketball arena, qualities such as speed and strength are crucial for a player development. The dynamic nature of the game demands that players make rapid and accurate decisions. Traditional practices focusing solely on speed or strength may not fully meet the developmental needs of athletes. Through diversified training methods, exploring various movement skills, players can cultivate agility in basketball across diverse scenarios. Delextrat^[23]^ suggests that small-sided games (SSG) in 2v2 training can improve the agility of basketball players (+4.5%, *P* < .05). SSG involves simulated training on a basketball court measuring 28 meters in length and 7.5 meters in half-width. SSG is considered an efficient and effective training method and is widely used by coaches to enhance physical fitness.^[24]^ The choice of 2v2 is based on the rationale that, compared to SSG involving more players, this type of training exhibits higher intensity and yields better training outcomes. Similarly, Zeng^[25]^ found that SSG can effectively enhance players’ shooting abilities, with greater benefits observed when using SSG before the start of the season. Combining SSG with dribbling training, agility training, or simulated games requires players to respond more flexibly to unexpected situations, promoting coordination and spatial awareness. The high-intensity, multidirectional, and variable-speed movements on the court enhance players’ explosiveness and agility,^[26]^ the fast-paced rhythm of compact games requires players to make quick decisions and reactions, cultivating their ability for rapid decision-making and response. From a physiological perspective, the high-intensity movements in SSG demand frequent explosive actions from players, prompting the cardiovascular system to efficiently supply oxygen and nutrients to muscle tissues. This contributes to enhancing athletes’ aerobic endurance and maintaining optimal agility within a short timeframe.^[27]^ In the fast-paced and dynamic environment of a basketball game, quick decision-making reflects a highly regulated nervous system. Continuous training through SSG enhances the responsiveness and coordination of the nervous system, thereby strengthening basketball agility.

**Figure 2.**
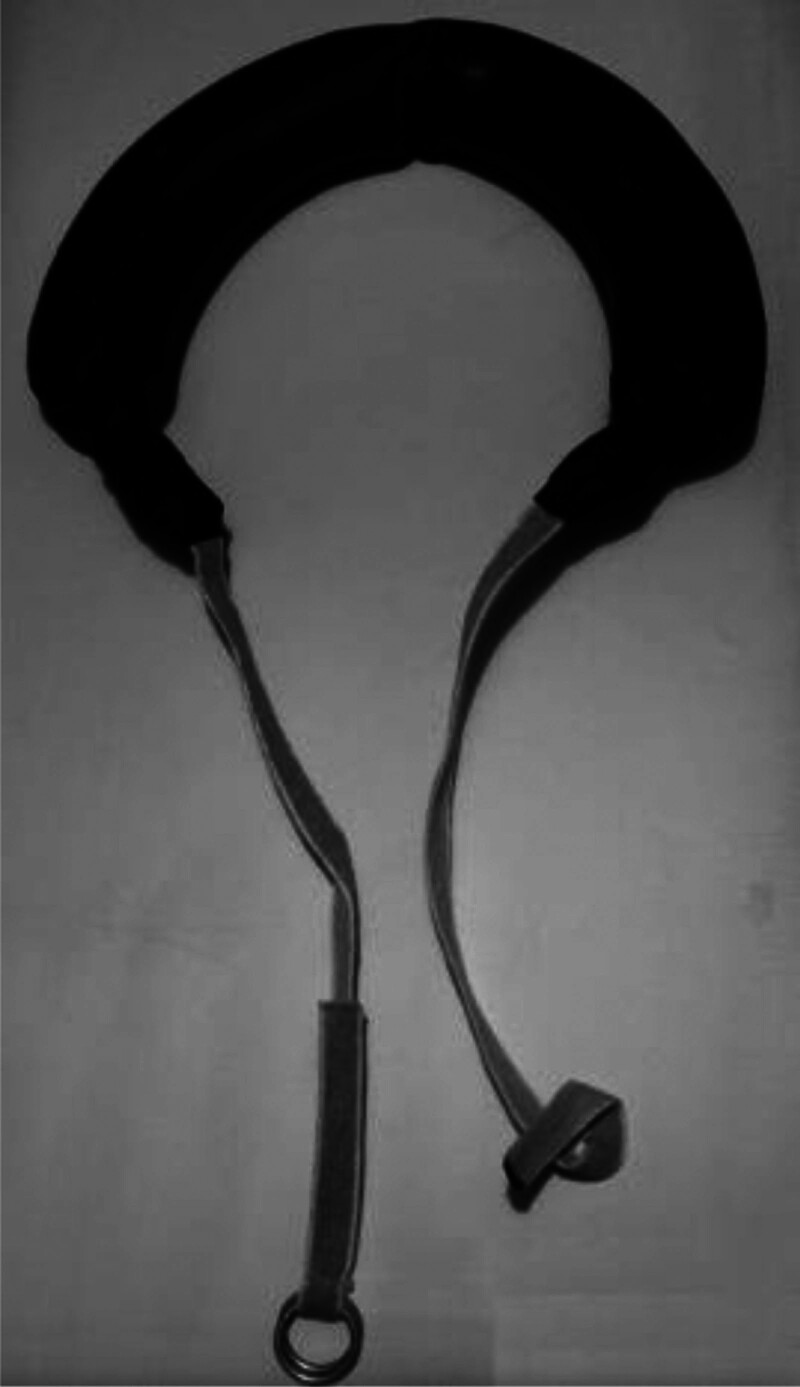
Bulgarian bag.

HANY^[28]^ suggests that an 8-week Bulgarian Bag training program can improve the agility of basketball players. The Bulgarian Bag was invented by a Bulgarian classical wrestler in 2005. It is a crescent-shaped exercise tool filled with sand, with weights ranging from 11 to 50 pounds (as shown in Fig. 2). The usage involves placing the bag around the athlete neck, with the ropes at both ends crossing in front of the chest. The bag is then secured between the athlete shoulder blades through the metal rings connected by the ropes.^[29]^ Due to the instability of its sand-filled structure, the Bulgarian sandbag engages more muscle groups and ligaments during use compared to iron equipment such as barbells and dumbbells, resulting in greater energy output. Additionally, it exhibits excellent adaptability and maneuverability during rotational, twisting, and squatting movements, making it more effective in enhancing the agility of basketball players. Özgür^[30]^ suggests that a 6-week TRX suspension training system can improve the agility of basketball players. TRX training utilizes suspended ropes and adjustable straps with handles, allowing various body exercises to be performed by suspending from fixed objects such as frames. The training leverages gravity and the suspension angle to provide intensity and difficulty, aiming to enhance core stability and muscle strength, thereby improving the agility of basketball players.

### 4.2. Research limitations and suggestions for future research

This study predominantly focused on professional and semiprofessional basketball players without age stratification within the study population. Consequently, the study outcomes may manifest distinct characteristics in agility development among athletes at different skill levels or age brackets, potentially constraining the generalizability of the findings. Additionally, despite utilizing established measures of agility, these tools may have limitations in comprehensively covering all relevant factors. The relatively brief duration of the study, centered around an approximately 8-week training period, may not fully capture the long-term changes in agility among basketball players. Furthermore, the study did not systematically consider the diversity of basketball training environments, encompassing various playing surfaces, climatic conditions, and other factors that could influence the generalizability of the results. Finally, individual differences, such as inherent talent and training responsiveness, along with other potential influencing factors, were not thoroughly explored. These limitations underscore the necessity for future research endeavors, including the adoption of more comprehensive samples, the development of innovative measurement tools, longitudinal tracking studies, and a more in-depth exploration of other potential factors influencing agility.

## 5. Conclusion

The application of enhanced training, comprehensive speed training, and diversified training methods (including SSG, TRX suspension training system, Bulgarian bag, etc) has shown significant potential to improve agility indicators in basketball players. These training methods effectively enhance basketball players’ performance in quick decision-making, rapid changes of direction, and high-intensity activities by improving muscle explosiveness, core stability, and neural adaptability. The adoption of comprehensive strength, speed, and agility training methods is expected to yield positive results in enhancing overall agility in athletes. However, it is worth noting that the effectiveness of these training methods may vary due to individual differences, the quality of training plan design, and execution. Therefore, personalized and systematic training programs are crucial for maximizing improvements in agility indicators for basketball players.

## Acknowledgments

We sincerely express our gratitude to all individuals and groups who contributed to this paper, especially those who provided professional writing services or material support. In particular, we would like to thank the corresponding author for providing professional writing support. Their expertise and advice played a crucial role in the development of this paper. Furthermore, our heartfelt thanks go to the Chengdu Sports University Library. Their contributions have provided valuable resources and perspectives, positively impacting our work.

We confirm that we have obtained permission from all individuals and groups mentioned in the acknowledgments section and have adhered to your editorial policies. Regarding the group authorship, we have included all authors in the title page and submission system. Please review our paper to ensure that the Acknowledgments section aligns with your editorial policies and authorship standards. If any additional information or modifications are required, we would be happy to accommodate. Once again, we appreciate your hard work and dedication.

## Author contributions

**Conceptualization:** Weicheng Li.

**Data curation:** Weicheng Li, Jiaxin Deng, Tong Wang.

**Funding acquisition:** Yongfeng Liu.

**Investigation:** Weicheng Li.

**Methodology:** Weicheng Li.

**Resources:** Yongfeng Liu.

**Supervision:** Yongfeng Liu.

**Visualization:** Weicheng Li.

**Writing – original draft:** Weicheng Li.

**Writing – review & editing:** Weicheng Li, Yongfeng Liu.
